# Psychometric testing of the Chinese version of the Perinatal Infant Care Social Support tool: a methodological study

**DOI:** 10.4069/whn.2024.05.21

**Published:** 2024-06-28

**Authors:** Feiyan Yi, Sukhee Ahn, Miyeon Park

**Affiliations:** 1 College of Nursing, Chungnam National University, Seoul, Korea; 2 Department of Nursing, Baekseok Culture University, Cheonan, Korea

**Keywords:** Infant care, Mothers, Primiparity, Social support, Tool translation

## Abstract

**Purpose:**

This study aimed to translate the Perinatal Infant Care Social Support (PICSS) instrument into Chinese and to verify the reliability and validity of the translated version.

**Methods:**

This study used a cross-sectional design to examine the reliability and validity of the Chinese version of the PICSS (C-PICSS). A cohort of 150 first-time mothers in China participated, attending hospital follow-up care at 6 weeks postpartum. Data were collected after obtaining informed consent from the mothers.

**Results:**

The majority of mothers were aged between 20 and 29 years, with a mean age of 26.25 (±3.90) years. An item analysis of the 19 items in the C-PICSS showed that all items had an item-total score correlation above 0.2. This resulted in a Kaiser-Meyer-Olkin value of 0.92 and a significant Bartlett’s test of sphericity (*χ*^2^=1,778.65, *p*<.001), confirming the suitability of the data for factor analysis. Correlation analyses revealed a strong positive relationship between infant care social support and general social support (r=.62, *p*<.001), and a negative relationship between infant care social support and postpartum depression (r=–.38, *p*<.001). Higher scores for infant care social support were associated with reporting positive relationships with their husbands (t=3.72, *p*<.001) and high levels of spousal involvement (t=4.09, *p*<.001). In terms of structural support, spouses were identified as the primary source.

**Conclusion:**

The research results indicate that C-PICSS is reliable and valid as an indicator of social support for infant care among Chinese mothers.

## Introduction

According to Mercer’s theory of becoming a mother, mothers acknowledge the changes that motherhood necessitates following childbirth. They actively seek information, identify role models, and become empowered to embrace the role of motherhood as part of an ongoing process [1]. Mothers engage in the practice of motherhood through interactions with family members, including their spouses and infants. They also continue to draw on the support of family, friends, and the wider community [2]. As mothers adapt to their new role, they focus on recovering their physical and mental health, fostering healthy interactions between themselves and their infants, as well as between themselves and their partners, and they learn the skills necessary for infant care [1].

First-time mothers often experience stress, anxiety, and a lack of confidence in their parenting abilities due to their inexperience with infant care as they navigate the uncharted territory of motherhood [3]. During this period, they require support from someone who can help them recognize their infant’s care needs—including nutrition, elimination, bathing, and responding to crying—and who can either teach them caregiving skills, assist with these tasks, or perform them as needed [4]. The amount and quality of postpartum social support are crucial for mothers to address their unmet needs, overcome parenting fears, build confidence in caring for their infants, and develop a maternal identity as they transition into motherhood [5,6]. Thus, there is a need for tools that can accurately measure the support related to infant care during the postpartum period.

A review of research on social support among first-time mothers in China found that a lack of social support made mothers’ role transition difficult, reduced satisfaction with their maternal role, and decreased their sense of parenting fulfillment, satisfaction, and happiness [7]. Difficulties in postpartum coping, stemming from inadequate infant care skills, were found to correlate negatively with postpartum social support [8]. Furthermore, studies have indicated that mothers experience higher levels of postpartum depression [7,8] and anxiety [9] when they perceive their social support to be lacking [9-11]. Conversely, higher social support has been linked to improved maternal role performance and easier adjustment to motherhood [12]. Social support specifically tailored to infant care has been demonstrated to strengthen first-time mothers’ confidence in caring for their newborns, facilitate their adaptation to motherhood, indirectly reduce postpartum depression, improve marital satisfaction, and positively influence sleep quality [13].

A review of instruments used internationally to measure social support among Chinese mothers identified a range of assessed support types, such as the kinds of support [8], sources of social support [9,14], and both the perceived importance and the actual support received [15]. Additionally, studies have measured objective support, subjective support, and the utilization of social support using instruments specifically designed for the Chinese context and culture [7,11].

The instruments previously discussed, however, have limitations in that they assess social support solely in terms of structure (resources) or function (types), without considering both. Moreover, these tools generally inquire about the frequency and resources of help but lack items specific to social support for infant care. The Perinatal Infant Care Social Support (PICSS) scale, which has been recently developed to measure social support for infant care, is tailored specifically for mothers in the perinatal setting and evaluates both functional and structural aspects of social support [16]. Functional support within this domain gauges the extent to which new mothers receive practical assistance, including informational support, as well as a network that offers encouragement, comfort, and appreciation for their baby care efforts. This could be a valid measure of social support for new mothers during the postpartum period, particularly in the context of infant care [16,17]. Structural support, in contrast, identifies who in the social network provides this support [16]. Consequently, this instrument is well-suited to assess the social support necessary for infant care, addressing both structural and functional aspects to facilitate mothers’ successful transition into motherhood.

Given the cultural differences in China, where mothers often depend on in-laws or biological parents for child-rearing support, as opposed to Western practices, it is crucial to adapt and refine instruments to ensure the adequacy of support resources and the relevance of infant care items for the Chinese context. Therefore, this study aimed to translate the PICSS, which measures both structural and functional support for infant care, into Chinese and to evaluate its validity.

This study aimed to conduct and evaluate psychometric testing of the Chinese version of the PICSS (C-PICSS). The specific purposes were as follows: (1) to translate the PICSS into Chinese, and (2) to verify its reliability and validity to employ it as a tool for measuring social support in infant care.

## Methods

Ethical Statement: This study was approved by the Institutional Review Board of Chungnam National University in Daejeon, Korea (202204-SB-050-01). Written informed consent was obtained from the research participants.

### Study design

This methodological study examined the reliability and validity of the C-PICSS, employing a cross-sectional survey. This study adhered to the STROBE (Strengthening the Reporting of Observational Studies in Epidemiology) guidelines [18].

### Tool translation procedure

The first author translated the original version of the PICSS [16] into Chinese after obtaining permission to use the tool from the PICSS developer. The Korean version of the PICSS [17], which has been translated and validated with Korean postpartum women, was selected for translation into Chinese, considering that the Chinese culture of parenting, such as receiving help from in-laws/biological parents, is different from that of the West and closer to that of Korea. The developer of the Korean version of the PICSS provided permission and consultation during the translation process. The original tool was also actively reviewed to ensure accuracy.

**Figure 1.**
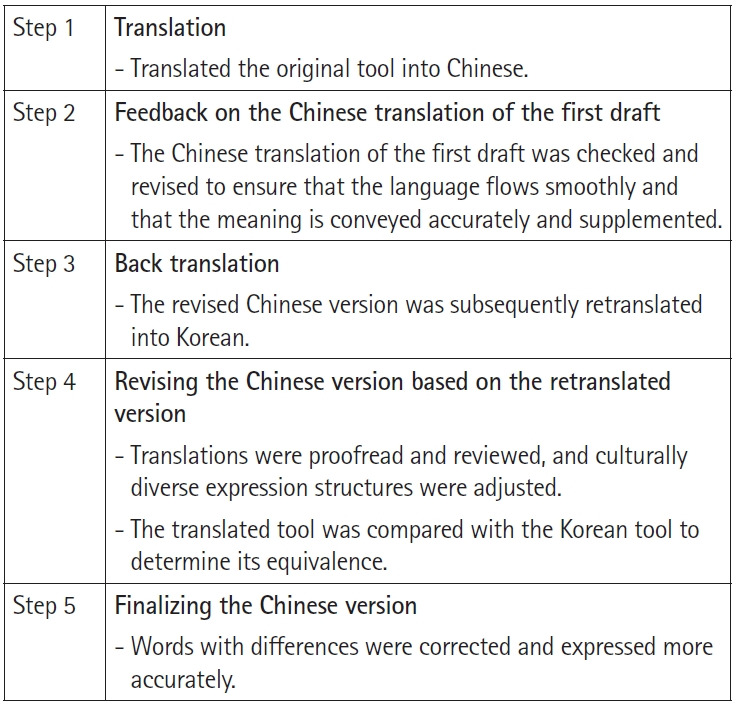
Development process of the Chinese version of Perinatal Infant Care Social Support scale.

The procedure for translating from Korean to Chinese followed five steps (Figure 1). In step 1, a graduate student bilingual in English and Chinese translated the first translation. In step 2, another bilingual graduate student reviewed the Chinese translation, providing feedback to ensure the language was smooth and the meaning was accurately conveyed. Improvements were made in collaboration with the initial translator. In step 3, a PhD student, fluent in both languages, independently back-translated the revised Chinese version into English. In step 4, the back-translated version and the original Korean instrument were reviewed by two professors specializing in women’s health nursing, who are proficient in both languages. They compared the two versions for inconsistencies arising from linguistic or cultural differences and provided suggestions for alternative words and expressions. In the final step, the first author and translator in steps 2 and 3 reviewed the Chinese translation for equivalence with the original instrument. During the comparison of the finalized version, any discrepancies in meaning were addressed by adjusting the wording to reflect the intended meaning more accurately. For instance, item 5, which originally read “Someone tells me that they are grateful to me,” was revised to “I have someone who expresses gratitude to me.” Subsequently, we evaluated the content validity of the translation by re-examining the meaning conveyed by each item, taking cultural and linguistic considerations into account. The feedback received confirmed that the translation was appropriate. Consequently, the C-PICSS was finalized.

### Participants

The study participants comprised 150 Chinese mothers at the Maternal and Child Health Hospital in Hebi City, Henan Province, China, who attended follow-up care at 6 weeks postpartum. The inclusion criteria were first-time mothers aged 20 years or older who were approximately 6 weeks postpartum, without any complications related to pregnancy, childbirth, or the postpartum period, and who were personally caring for their infants at home. The exclusion criteria were women with an infant who was currently hospitalized or cared for by someone other than the mothers.

The researcher (first author) posted a recruitment notice on the outpatient bulletin board of the maternity department at a tertiary hospital, located in Hebi, a large Chinese city in Henan Province. Interested mothers were invited to approach the researcher for more information. The researcher then explained the study’s purpose and methods to these mothers, assessed their eligibility, and presented an informed consent form to those who qualified. Mothers who chose to participate voluntarily signed an informed consent form.

Based on a minimum of 5 to 10 participants per item required for tool development [19], considering the 19 items of this instrument, a minimum of 95 and a maximum of 190 participants were deemed necessary. Therefore, 150 participants were recruited for this study.

### Instrument

The finalized version of the 19-item C-PICSS assesses the functional and structural social support mothers require for infant care. The original PICSS functional support domain includes a support system and practical support [16]. In contrast, the Korean version of Perinatal Infant Care Social Support (K-PICSS) [17] is divided into three domains: informational support, support system, and practical support. Responses to each item range from 1, indicating “never,” to 5, indicating “always,” with higher scores (possible range, 19–95) denoting greater levels of social support. The reliability of the original instrument was demonstrated with a Cronbach’s α of .90 for the support system domain and .86 for the practical support domain [16]. The K-PICSS [17] showed subscale reliability values between .82 and .83, and the overall reliability of the instrument was .90. A Cronbach’s α for the K-PICSS overall was .90. The validity of the instrument has been confirmed as well.

In the structural support domain, doctors and nurses are categorized as professional sources of support, while husbands, parents-in-law, parents, sisters, friends, neighbors, and the internet are considered nonprofessional sources. Structural support involves identifying the sources of support for each item and subsequently evaluating the frequency and percentage of utilization for each source.

Construct validity was evaluated through exploratory factor analysis, correlation analysis, and known-group comparisons. For known-group comparison, the following general characteristics were assessed: maternal age, occupation, education, family monthly income, economic level, relationship with husband, husband’s involvement in postpartum care, maternal health status, and the baby’s health status. To evaluate the concurrent validity of the C-PICSS, its correlations with social support and postpartum depression were assessed.

#### Postpartum social support

To measure the concurrent validity of the C-PICSS, we utilized the Chinese version [15] of the Postpartum Social Support Questionnaire (PSQ) [15]. Comprising 34 items, the PSQ is divided into four domains: material support, emotional support, informational support, and peer support. Respondents rate each item on a 7-point scale, where 1 signifies no support, and 7 indicates a high level of support. Higher scores (possible range, 34–238) reflect greater satisfaction with the received social support. This questionnaire has a reliability coefficient of .94 and a content validity index of .96. In our study, the reliability of the PSQ was confirmed with a Cronbach’s α of .96.

#### Postpartum depression

The Chinese version of the Edinburgh Postnatal Depression Scale (EPDS) [20] was utilized to measure the concurrent validity of the C-PICSS. The EPDS consists of 10 items, each rated on a 4-point scale, with item scores ranging from 0 to 3 (possible range, 0–30). The threshold for depression is set at a score of 9/10 in China [21], with scores from 0 to 9 considered normal and scores of 10 or higher indicating a risk for depression. Higher scores suggest more severe depressive symptoms. The reliability was reported with a Cronbach’s α of .76, and its content validity was .93. In this study, the instrument’s reliability was demonstrated by a Cronbach’s α of .86.

### Data collection

The data collection period spanned from October 24 to December 19, 2022. The researcher outlined the study’s objectives and methods to the outpatient nurses responsible for obstetrics and gynecology at the Maternal and Child Health Hospital in Hebi City, Henan Province, China, requesting their assistance with the research. The first author posted subject recruitment flyers on hospital notice boards; potential participants visited the first author to verify eligibility and explain the aims and methods of the study. Once the mothers consented to participate, they filled out the study questionnaire once, which took approximately 15 minutes, and then returned it to the researcher. As a token of appreciation for their participation, the mothers were given baby wipes as a gift.

### Data analysis

The collected data were analyzed using IBM SPSS ver. 26.0 (IBM Corp., Armonk, NY, USA), and the statistical significance level was set at less than 5%. Exploratory factor analysis was performed to assess the construct validity of the C-PICSS. To evaluate the concurrent validity of the C-PICSS, correlation analysis was carried out with measures of postpartum social support and depression. Construct validity was further assessed through comparisons between known groups. Reliability testing included item analysis and assessment of the instrument’s consistency in measuring the phenomenon. Differences in infant care social support according to participants’ characteristics were examined using the t-test or one-way analysis of variance.

## Results

### Characteristics of participants

Most mothers (80.0%) were between 20 and 29 years old, with a mean age of 26.25 (±3.90) years. Of these mothers, 37.3% were employed and 48.7% were college graduates or higher. Most (87.3%) had a monthly household income of 3,000 Chinese yuan (approximately 415 US dollars) or more, which corresponds to middle class and above based on 3,073 yuan as a monthly average income in 2022 statistics [22], and 65.3% reported middle-class economic status. Two-thirds of mothers (66.7%) delivered vaginally, 93.3% reported that they were in good health, and 84.0% perceived their infants as being in good health. Three-fourths of mothers (76.0%) reported a good relationship with their husbands, and 72.7% indicated a high level of husband’s involvement in postpartum care (Table 1).

### Construct validity

Item analysis of the 19 items in the C-PICSS revealed that all item-total correlations were above 0.3, so all items were included in the factor analysis. Exploratory factor analysis confirmed construct validity, with a Kaiser-Meyer-Olkin value of 0.92 and a Bartlett’s test of sphericity yielding *χ*^2^=1,778.65 (*p*<.001), indicating the data were appropriate for factor analysis. Commonality scores ranged from a minimum of .51 to a maximum of .74, all exceeding the threshold of 0.4. Utilizing principal component analysis, an eigenvalue cutoff of 1 or higher, and varimax rotation, three factors emerged. The eigenvalues for these factors were 4.14, 4.06, and 3.92, with the respective explanatory powers being 21.80%, 21.39%, and 20.64%, yielding a total explanatory power of 63.83%.

After checking the properties of the three factors in the C-PICSS, we assigned names to each. Factor 1 was termed “practical support,” as it contained items evaluating the mother’s receipt of tangible assistance, including household chores, childcare, and bathing. Factor 2 was designated “informational support,” reflecting its focus on providing information to mothers regarding infant care practices such as bathing, feeding, and dressing. Factor 3 was labeled “support system” because its elements are related to various sources of support systems to meet mothers’ infant care needs. The reliability of the subscales was high, with Cronbach’s α values ranging from .87 to .89, and the overall reliability of the instrument was shown by a Cronbach’s α of .93 (Table 2).

### Concurrent validity

The correlations (r) among the three subdomains of infant care social support ranged from .85 to .92 (*p*<.001). The C-PICSS demonstrated a positive moderate correlation with social support in the postpartum period (r=.62, *p*<.001) and exhibited a negative weak correlation with postpartum depression (r=–.38, *p*<.001) (Table 3).

### Known-group comparison

To test the instrument’s validity using a comparison group, we investigated differences in C-PICSS scores according to the relationship with the husband and the level of the husband’s involvement in postpartum care. Participants who reported a good relationship with their husbands had higher C-PICSS scores than those with a poor relationship (t=3.72, *p*<.001). Similarly, scores were higher for participants whose spouses were highly involved in postpartum care than for those with spouses who were less involved (t=4.09, *p*<.001) (Table 4).

### Levels of C-PICSS: structural and functional support

The results for structural and functional support, as shown by the infant care social support items, were evaluated. In the structural support assessment, the spouse was the first choice for practical support, followed by parents-in-law and parents. Regarding informational support, friends ranked first, followed by spouses, doctors, and parents-in-law. In the support system category, spouses were the most common, followed by friends and parents. Overall, the spouse was the primary source of support.

In the functional support assessment, participants’ scores were as follows: 21.08 (±5.45) for practical support (item mean, 3.27–3.64), 20.82 (±5.22) for information support (item mean, 3.34–3.64), and 24.92 (±6.70) for the support system (item mean, 3.04–3.64), for a total score of 66.83±15.57 (Table 5).

### Differences in C-PICSS by participant characteristics

Mothers with a job (t=3.30, *p*=.001), college or higher education (t=4.39, *p*=.014), high family monthly income (t=3.12, *p*=.028), and moderate economic status (F=9.36, *p*<.001) had higher social support for infant care than their counterparts. Furthermore, the infant care social support scores were higher if the baby was in good health (t=2.12, *p*=.035) (Table 1).

## Discussion

This study translated the original PICSS into Chinese to develop the C-PICSS. It determined the reliability and validity of this instrument, which measures the social support Chinese mothers receive when caring for their postpartum infants. The findings indicated that the C-PICSS is a reliable and valid tool for evaluating social support for infant care among Chinese mothers.

This study adhered to established guidelines for instrument translation to maintain the content validity of the original tool. Throughout the translation phase, professors specializing in women’s health nursing and Chinese international nursing students at the graduate level scrutinized the translated version for linguistic accuracy and semantic integrity, ensuring its cultural adaptation to the Chinese context and fidelity to the linguistic environment. During data collection, participants evaluated each item on the C-PICSS instrument regarding functional and structural support resources. Since the Chinese mothers comprehended and responded to all 19 items, the C-PICSS item expressions appear to be congruent with Chinese culture and the linguistic environment.

The construct validity of the C-PICSS was established through exploratory factor analysis, correlation analysis, and known-group comparisons. The exploratory factor analysis revealed three subdomains of functional support: practical support, informational support, and support system. These subdomains align with the three identified in the K-PICSS [17]. Regarding structural support, spouses and friends were identified as primary sources, while in-laws and parents were also commonly utilized for support. This pattern mirrors the findings of a Korean study [17]. It likely reflects the similarities in mothers’ infant care practices, support sources, and support needs across Asian cultures, as opposed to Western cultures.

The practical support domain of infant care includes assistance with care needs such as sharing household chores, providing infant care support, and helping with bathing. These forms of assistance are key facilitators for mothers adjusting to their new role [16]. When Chinese mothers received emotional support and practical help with infant care from family, friends, or professionals, it helped them adapt to stress and emotional challenges during the postpartum period, reducing the incidence of postpartum depression and coping difficulties [7,8]. Furthermore, in Chinese mothers, receiving higher levels of support regarding parenting information and postpartum care needs, including bathing, feeding, and dressing their infants, was associated with fewer postpartum coping difficulties [8]. For Chinese mothers, a support system that offered attention, encouragement, comfort, and positive feedback strengthened their confidence in fulfilling their maternal roles. This led to improved role performance and greater parental satisfaction [12]. These findings indicate that, besides practical and informational support, encouraging, positive verbal reinforcement and supportive attitudes are important factors in adjusting to motherhood.

Mothers were found to utilize multiple sources of support to meet their infant care needs. For practical support, spouses were the primary source, followed by in-laws and the mothers’ own parents. Similarly, in emotional support, spouses were again the main providers, with friends and parents also playing significant roles. This aligns with a study of Korean mothers [17], which indicated that husbands were the most frequent source of support across both domains. Additionally, traditional postpartum care customs are still prevalent in Chinese families, with the majority of postpartum care occurring at home. Here, spouses, parents-in-law, and other relatives play key roles in supporting the mother’s postpartum needs. Conversely, regarding informational support, friends were the most common source, followed by spouses. This contrasts with the findings from a study on Korean mothers [17], where parents were the main sources, followed by friends and spouses. This discrepancy may stem from China’s extended family structure instead of Korea’s nuclear family orientation. Korean mothers may depend more on their parents for information, even if they live far away. In contrast, the younger average age of Chinese mothers in this study (26 years) suggests they may be more inclined to leverage the convenience of information sharing and acquisition through smartphones. Chatting with friends allows them to access parenting and postpartum care information readily. The opportunity for mothers to share their experiences, gain reassurance, and feel understood during the significant life transition of the perinatal period is greatly enhanced by the support of friends, particularly those who are also mothers [23].

In China, hospitals provide telephone counseling to mothers on postpartum day 7, and mothers visit the hospital for postpartum checkups on postpartum days 14 and 42. Despite these multiple opportunities for healthcare providers to provide postpartum information to mothers, they were ranked last as sources of information support. This suggests that healthcare providers are not effectively meeting the needs of mothers seeking specialized parenting information.

Receiving less social support from healthcare professionals has been identified as a predictor of postpartum depression [24]. Conversely, advice from nursing professionals is the most significant factor associated with social support from these professionals [25]. These studies underscore the critical role of healthcare providers in forming supportive partnerships with postpartum mothers. Consequently, it is essential for healthcare providers to be equipped to share their knowledge on postpartum care and parenting effectively. They should also employ strategies to enhance the availability of parenting information, such as distributing hospital mailings and providing links to informational resources or videos on smartphones for mothers.

Spouses were the most important source of both structural and functional support, and social support scores were significantly higher when mothers had a good relationship with their husbands and when their husbands were more involved in the postpartum period. The presence of supportive partners, including husbands or other partners, can serve a protective function by assisting the new mother in adapting to her role and managing any physical or mental health challenges that may arise after childbirth [26]. Furthermore, there was a negative correlation between social support for infant care and postpartum depression, reinforcing the idea that a higher level of maternal social support is linked to a decrease in postpartum depression [9,23,24]. This suggests that improving the mother-spouse relationship could be an effective approach to actively involve them as a source of social support for infant care.

This study demonstrated reliability and validity for the C-PICSS, showing that it can be used to assess social support for infant care among Chinese mothers. However, a limitation of this study is its exclusion of smartphone applications and social network services as potential internet sources of social support, thereby failing to capture the use of smartphone-accessible resources. Korean mothers frequently utilize smartphones to access web and mobile sites for parenting and baby care information [17]. Similarly, in China, mothers can search for keywords on platforms such as Baidu, Sina, and Tencent to find extensive parenting and postpartum care information presented through videos, photos, and text [10]. Future research should, therefore, incorporate a range of digital resources into the concept of structural support to assess their use more comprehensively. Additionally, since this study only enrolled first-time mothers, it is important to investigate whether this social support tool is also applicable to mothers with multiple children. It is also essential to identify the facilitators and barriers to social support and to pursue further research on strategies to enhance social support and secure supportive resources, which will assist mothers in adjusting to motherhood.

## Figures and Tables

**Table 1.** Differences in Perinatal Infant Care Social Support by participant characteristics (N=150)

Variable	Categories	n (%)	Mean±SD	t/F (*p*) Scheffé
Age (year)			26.25±3.90	
	20–29	120 (80.0)	67.06±14.99	–0.36 (.715)
	≥30	30 (20.0)	65.90±17.94	
Job	Yes	56 (37.3)	72.10±15.28	3.30 (.001)
	No	94 (62.7)	63.69±14.95	
Level of education	Primary or Secondary school^a^	34 (22.6)	62.41±17.52	4.39 (.014)
	High school or vocational school^b^	43 (28.7)	63.97±13.79	a<c
	University and above^c^	73 (48.7)	70.57±14.89	
Monthly household income (CNY)	<3,000	19 (12.7)	60.78±19.95	3.12 (.028)
	3,000–5,000	48 (32.0)	65.85±14.11	
	5,000–8,000	42 (28.0)	65.14±16.63	
	>8,000	41 (27.3)	72.51±12.33	
Economic level perceived	High^a^	3 (2.0)	69.33±10.06	9.36 (<.001)
	Middle^b^	98 (65.3)	70.50±15.09	b>c
	Low^c^	49 (32.7)	59.34±14.25	
Type of delivery	Vaginal birth	100 (66.7)	68.47±15.75	1.83 (.069)
	Cesarean birth	50 (33.3)	63.56±14.81	
Maternal health status	Good	140 (93.3)	67.22±15.67	1.16 (.246)
	Not good	10 (6.7)	61.30±13.62	
Infant’s health status	Good	126 (84.0)	68.00±15.15	2.12 (.035)
	Not good	24 (16.0)	60.70±16.60	
Relationship with husband	Good	114 (76.0)	69.38±15.13	3.72 (<.001)
	Moderate/Poor	36 (24.0)	58.75±14.30	
Husband’s involvement in postpartum care	High	109 (72.7)	69.87±14.94	4.09 (<.001)
	Low	41 (27.3)	58.75±14.42	

CNY: Chinese Yuan (5,000 CNY is roughly 692 US dollars).

**Table 2.** Factor analysis of the Chinese version of the Perinatal Infant Care Social Support scale (N=150)

Items	Factor		
	Practical support	Informational support	Support system
8. I won’t be on my own taking care of my infant.	.80	.10	.22
我不用自己独自一个人照顾孩子			
12. I can get hands-on help with comforting my infant.	.75	.34	.31
我哄孩子的时候可以得到直接的帮助			
9. I have someone to help with routine housework.	.72	.22	.24
我有帮我做家务的人			
13. I can get hands-on help with infant changing/dressing.	.72	.30	.35
我在给孩子更换衣服的时候可以得到直接的帮助			
17. I can get hands-on help with infant bathing.	.67	.30	.05
我给孩子洗澡的时可以得到直接帮助			
14. I can get hands-on help with infant feeding.	.50	.39	.40
我可以在哺乳孩子方面获得直接的帮助			
19. I can get information on taking care of my body after birth. 我可以获得关于产后调理的相关信息（比如身体的管理）	.14	.80	.22
18. I can get consistent information on infant care.	.23	.76	.17
我可以获得有关育儿的一致信息			
16. I can get information on infant bathing.	.28	.73	.12
我可以获得有关婴儿沐浴的相关信息			
15. I can get information on infant feeding.	.32	.70	.20
我可以获得哺乳的相关信息			
10. I can get information on infant comfort/settling.	.34	.57	.25
我可以获得哄孩子的相关信息			
11. I can get information on infant changing/dressing.	.47	.54	.22
我可以获得给孩子更换衣服的相关信息			
5. I have someone who shows me appreciation.	.18	.09	.75
有人向我表达谢意			
4. I have someone to talk to about how I feel.	.20	.29	.75
我有可以倾诉情绪/倾诉感受的人			
3. Those close to me understand that it is ok for me to need help. 我身边的人都理解，我需要帮助是理所当然的	.15	.28	.73
1. I have someone to care for and comfort me.	.34	.11	.72
我有照顾和安慰我的人			
6. If I need advice there is someone who will assist me.	.28	.30	.62
我需要建议的时候，有可以给我提供帮助的人			
7. I have people to count on when things go wrong.	.50	.14	.55
即使我做的不好，也有可以依靠的人			
2. I have someone to talk to and share experiences with.	.00	.59	.53
我有可以与之交谈和分享经验的人			
Eigenvalue	4.14	4.06	3.92
Explained variance (%)	21.80	21.39	20.64
Cumulated variance (%)	21.80	43.19	63.83
Range of item-total correlation	.62–.82	.66–.71	.59–.71
Cronbach’s α for subscales	.89	.87	.87
Cronbach’s α for C-PICSS	.93		

**Table 3.** Relationships among Perinatal Infant Care Social Support, social support, and postpartum depression (N=150)

Variable	r (*p*)					
	1	2	3	4	5	6
1. Infant care social support_Total	1					
2. Infant care social support_Information support	.85 (<.001)	1				
3. Infant care social support_Support system	.92 (<.001)	.68 (<.001)	1			
4. Infant care social support_Practical support	.89 (<.001)	.64 (<.001)	.76 (<.001)	1		
5. Social support	.62 (<.001)	.55 (<.001)	.60 (<.001)	.49 (<.001)	1	
6. Postpartum depression	–.38 (<.001)	–.46 (<.001)	–.30 (<.001)	–.26 (<.001)	–.29 (<.001)	1

**Table 4.** Comparison of scores of the Chinese version of the Perinatal Infant Care Social Support scale according to relationship with husband and the husband’s involvement in postpartum care (N=150)

Variable	Categories	n (%)	Mean±SD	t (*p*)
Relationship with husband	Good	114 (76.0)	69.38±15.13	3.72(<.001)
	So so/ not good	36 (24.0)	58.75±14.30	
Husband’s involvement in postpartum care	High	109 (72.7)	69.87±14.94	4.09 (<.001)
	Low	41 (27.3)	58.75±14.42	

**Table 5.** Levels of Chinese Perinatal Infant Care Social Support scale: Structural and Functional Support (N=150)

Factor	Item	Structural support, n (%)								Functional support, Mean±SD
		Husband	Parents- in-law	Parents	Sister	Friend	Neigh-bor	Doctor	Nurse	
Practical support	8. I won’t be on my own taking care of my infant.	57 (38.0)	49 (32.7)	29 (19.3)	3 (2.0)	4 (2.7)	3 (2.0)	1 (0.7)	4 (2.7)	3.62±1.37
	12. I can get hands-on help with comforting my infant.	63 (42.0)	36 (24.0)	26 (17.3)	4 (2.7)	7 (4.7)	1 (0.7)	7 (4.7)	6 (4.0)	3.46±1.23
	9. I have someone to help with routine housework.	64 (42.7)	49 (32.7)	19 (12.7)	5 (3.3)	6 (4.0)	3 (2.0)	2 (1.3)	2 (1.3)	3.56±1.31
	13. I can get hands-on help with infant changing/ dressing.	69 (46.0)	38 (25.3)	20 (13.3)	2 (1.3)	2 (1.3)	5 (3.3)	6 (4.0)	8 (5.3)	3.46±1.27
	17. I can get hands-on help with infant bathing.	69 (46.0)	29 (19.3)	15 (10.0)	4 (2.7)	7 (4.7)	2 (1.3)	10 (6.7)	14 (9.3)	3.64±1.26
	14. I can get hands-on help with infant feeding,	56 (37.3)	18 (12.0)	16 (10.7)	4 (2.7)	16 (10.7)	3 (2.0)	21 (14.0)	16 (10.7)	3.27±1.29
Informational support	19. I can get information on taking care of my body after birth.	26 (17.3)	10 (6.7)	3 (2.0)	6 (4.0)	23 (15.3)	6 (4.0)	52 (34.7)	24 (16.0)	3.34±1.23
	18. I can get consistent information on infant care.	25 (16.7)	25 (16.7)	10 (6.7)	9 (6.0)	28 (18.7)	9 (6.0)	25 (16.7)	19 (12.7)	3.60±1.08
	16. I can get information on infant bathing.	27 (18.0)	14 (9.3)	10 (6.7)	12 (8.0)	37 (24.7)	4 (2.7)	22 (14.7)	24 (16.0)	3.64±1.12
	15. I can get information on infant feeding.	29 (19.3)	15 (10.0)	19 (12.7)	9 (6.0)	21 (14.0)	7 (4.7)	31 (20.7)	19 (12.7)	3.58±1.16
	10. I can get information on infant comfort/settling.	25 (16.7)	26 (17.3)	21 (14.0)	12 (8.0)	37 (24.7)	8 (5.3)	13 (8.7)	8 (5.3)	3.58±1.10
	11. I can get information on infant changing /dressing.	32 (21.3)	35 (23.3)	26 (17.3)	9 (6.0)	22 (14.7)	4 (2.7)	10 (6.7)	12 (8.0)	3.63±1.02
Support system	5. I have someone who shows me appreciation.	86 (57.3)	8 (5.3)	9 (6.0)	3 (2.0)	31 (20.7)	6 (4.0)	3 (2.0)	4 (2.7)	3.04±1.25
	4. I have someone to talk to about how I feel.	71 (47.3)	2 (1.3)	9 (6.0)	14 (9.3)	46 (30.7)	6 (4.0)	2 (1.3)	0 (0)	3.60±1.18
	3. Those close to me understand that it is ok for me to need help.	61 (40.7)	15 (10.0)	29 (19.3)	11 (7.3)	24 (16.0)	8 (5.3)	1 (0.7)	1 (0.7)	3.46±1.13
	1. I have someone to care for and comfort me.	90 (60.0)	10 (6.7)	28 (18.7)	9 (6.0)	6 (4.0)	2 (1.3)	0 (0)	5 (3.3)	3.64±1.09
	6. If I need advice there is someone who will assist me.	65 (43.3)	9 (6.0)	21 (14.0)	7 (4.7)	31 (20.7)	7 (4.7)	6 (4.0)	4 (2.7)	3.52±1.06
	7. I have people to count on when things go wrong.	93 (62.0)	4 (2.7)	37 (24.7)	4 (2.7)	5 (3.3)	4 (2.7)	2 (1.3)	1 (0.7)	3.58±1.21
	2. I have someone to talk to and share experiences with.	27 (18.0)	3 (2.0)	14 (9.3)	19 (12.7)	73 (48.7)	8 (5.3)	4 (2.7)	2 (1.3)	3.55±1.03
Total										66.83±15.57
